# Nonrandom Distribution of Vector Ticks (*Dermacentor variabilis*) Infected by *Francisella tularensis*

**DOI:** 10.1371/journal.ppat.1000319

**Published:** 2009-02-27

**Authors:** Heidi K. Goethert, Sam R. Telford

**Affiliations:** Division of Infectious Diseases, Cummings School of Veterinary Medicine, Tufts University, North Grafton, Massachusetts, United States of America; National Institute of Allergy and Infectious Diseases, United States of America

## Abstract

The island of Martha's Vineyard, Massachusetts, is the site of a sustained outbreak of tularemia due to *Francisella tularensis tularensis*. Dog ticks, *Dermacentor variabilis*, appear to be critical in the perpetuation of the agent there. Tularemia has long been characterized as an agent of natural focality, stably persisting in characteristic sites of transmission, but this suggestion has never been rigorously tested. Accordingly, we sought to identify a natural focus of transmission of the agent of tularemia by mapping the distribution of PCR-positive ticks. From 2004 to 2007, questing *D. variabilis* were collected from 85 individual waypoints along a 1.5 km transect in a field site on Martha's Vineyard. The positions of PCR-positive ticks were then mapped using ArcGIS. Cluster analysis identified an area approximately 290 meters in diameter, 9 waypoints, that was significantly more likely to yield PCR-positive ticks (relative risk 3.3, P = 0.001) than the rest of the field site. Genotyping of *F. tularensis* using variable number tandem repeat (VNTR) analysis on PCR-positive ticks yielded 13 different haplotypes, the vast majority of which was one dominant haplotype. Positive ticks collected in the cluster were 3.4 times (relative risk = 3.4, P<0.0001) more likely to have an uncommon haplotype than those collected elsewhere from the transect. We conclude that we have identified a microfocus where the agent of tularemia stably perpetuates and that this area is where genetic diversity is generated.

## Author Summary

We present evidence, for the first time, that the agent of tularemia persists in microfoci for at least four years. The existing literature alludes to the natural nidality of this bacterium and the importance thereof in its long-term survival in nature, but “natural foci” that have been described to date have been very nebulously defined, poorly analyzed, and at a scale much larger than what we describe. In addition, we demonstrate such a focus by using modern GIS methods as well as by genetic cluster analysis of bacterial DNA. The work, therefore, contributes to our understanding of how the agent of tularemia perpetuates over the long term. Furthermore, this paper serves as a paradigm for analyzing the ecology of other vector-borne infections and, in particular, demonstrating the mode of their long-term persistence in the environment.

## Introduction

Martha's Vineyard, Massachusetts, is the site of a sustained outbreak of tularemia, due to *Francisella tularensis tularensis* [1], starting in 2000 and persisting through 2008. Although ulceroglandular disease is the most commonly reported form of tularemia in the United States, the majority of the more than 70 cases identified thus far on Martha's Vineyard have presented with the pneumonic form of the disease. A large proportion of the patients worked as landscapers; a case control study implicated lawn mowing and brush cutting as high risk activities [2], but the nature of the fomites remains undescribed. Exposure to aerosols generated from rabbits or their carcasses does not appear to explain this outbreak. Interestingly, the only other outbreak of aerogenic tularemia in the United States was reported from Martha's Vineyard in 1978 [3]. No defined outbreak or epizootic was recognized on Martha's Vineyard in the interim between the two episodes of pneumonic transmission even though solitary cases have been reported [1]. Where the agent was maintained during this interepizootic/interepidemic period remains undescribed. Understanding the local determinants of *F. tularensis* transmission would help us to understand interepizootic maintenance as well as help to identify the fomites that are presumably the basis of aerogenic risk.

We have identified American dog ticks (*Dermacentor variabilis* herein referred to as dog tick) as an important if not the major factor in perpetuation of *F. tularensis tularensis* on Martha's Vineyard. Prevalence of *F. tularensis* DNA in dog ticks collected from sites throughout the island ranges from 1% to 5%. Using variable number tandem repeat (VNTR) analysis, we demonstrated that the diversity of *F. tularensis tularensis* in dog ticks from Martha's Vineyard is as great as that measured for all existing *F. tularensis* isolates from across North America [4]. This suggests that the agent of tularemia has been endemic on Martha's Vineyard since its likely introduction in the 1930s and argues against periodic reintroduction events by migratory birds infested with *F. tularensis* infected ticks. Our observations strongly suggest that the agent of tularemia has been maintained continuously but cryptically in between the 1978 and 2000 outbreaks and we thus seek to identify the sites and mode of such persistence. In addition, how great genetic diversity is generated and maintained within an island of 232 square kilometers remains to be explored.

The theory of the natural nidality/focality of vector-borne infections (also known as landscape epidemiology) provides a conceptual basis for explaining the long-term persistence of tularemia on Martha's Vineyard. Zoonotic agents are maintained in nature in nidi, or foci, of transmission comprising characteristic associations of pathogen, fauna and flora (“pathobiocenose”). The proposed nidi could range from as small as a single rodent hole (“microfocus”) to as large as a landscape zone, such as a river floodplain or taiga forest [5],[6]. Permanent foci (“elementary foci”) exist where there is a longstanding, undisturbed pathobiocenose; such foci serve as an environmental reservoir from which other foci may be initiated due to spillover during periods of amplification. The perpetuation of diverse infections, such as plague [7], rabies [8], and tick-borne encephalitis [9] appear to be best explained by natural nidality. Tularemia has been classified as an infection of natural nidality [5],[10] but rigorous tests of this assertion have not been presented. It may be that *F. tularensis tularensis* is maintained on Martha's Vineyard as a metapopulation of small cryptic foci. Individual microfoci might be isolated enough to allow genetic drift to occur and thereby explain the great genetic diversity of *F. tularensis* that we detect throughout the island. Accordingly, we sought to identify a microfocus of tularemia on Martha's Vineyard by finely mapping within an enzootic site the distribution of ticks containing DNA of *F. tularensis*. In addition, we determined whether genetic diversity of *F. tularensis* may be nonrandomly distributed in our study site.

## Results

**Figure 1:**
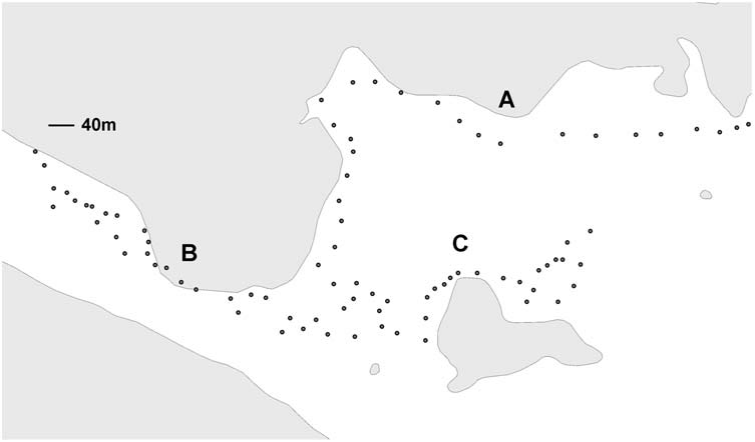
Map of the field site with three transects and 85 individual waypoints.

Dog ticks were collected monthly for 4 years (2004–2007) from 85 waypoints on three transects (A–C) in our field site near Squibnocket (Figure 1). A minimum of 1500 ticks was examined each year (Table 1). The annual prevalence of dog ticks testing positive by PCR for *F. tularensis* DNA ranged from 2.7% to 4.3% with a median of 3.4% (Table 1). However, there was no significant difference between the prevalence from year to year, inasmuch as all the confidence intervals overlap.

**Table 1:** Prevalence of the agent of tularemia in *Dermacentor variabilis*.

Year	Number	Prevalence [95% CI]	Population Index*
2004	1,522	2.8% [2.1, 3.8]	11.8
2005	2,103	4.3% [3.5, 5.2]	22.8
2006	1,611	3.6% [2.7, 4.6]	20.8
2007	1,932	2.7% [2.1, 3.6]	16.8

*: Average number of ticks collected per 10m in June.

**Figure 2:**
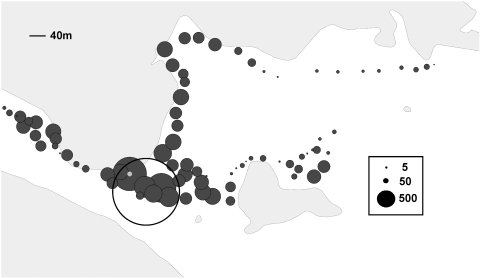
GIS mapping of the total number of *Dermacentor variabilis* collected at each waypoint, 2004–2007. The size of the dot at each waypoint is proportional to the number of ticks at each site. The location of the cluster identified by SaTScan is circled. Waypoint 70 is indicated by the gray dot.

The number of ticks collected from any given waypoint varied each year, and the distribution of ticks was not constant across the field site. Figure 2 shows the total number of ticks collected from each waypoint over the course of the study. In 2004, the majority of ticks were collected along transect B. However, by 2007, part of this transect was often flooded and very few ticks were collected there. Most of the ticks for the 4 year study were collected along the western section of transect A as well as the area where the three transects intersect, with the eastern portion of transects A and C yielding very few ticks. The western portion of the site generally yielded more ticks; however the year-to-year numbers were inconsistent (data not shown). The intersection of the 3 transects was the only area which consistently yielded large numbers of ticks every year; cumulatively, the greatest number of ticks for the entire study was obtained from this portion of the transect. Thus, ticks nonrandomly clustered along our transect.

**Figure 3 ppat-1000319-g003:**
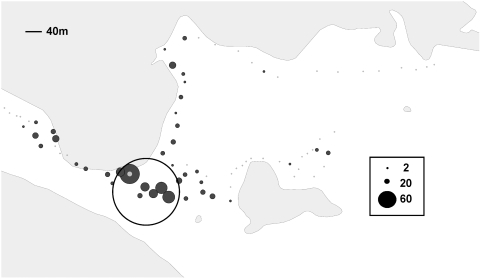
GIS mapping of the total number of ticks that tested positive for *Francisella tularensis tularensis* DNA by PCR from each waypoint. The size of the dot at each waypoint is proportional to the number of ticks at each site. The location of the cluster identified by SaTScan is circled. Waypoint 70 is indicated by the gray dot.

PCR-positive ticks (Figure 3) could be found anywhere along the transect where sufficient numbers of ticks were collected, but the intersection of the transects yielded the greatest proportion of positives. To determine whether the greater number of PCR-positive ticks found at the intersection of the transects was simply due to the fact that we collected a large number of ticks there, we performed a cluster analysis using SaTScan. SaTScan identified the area of intersection (circled on Figures 2–4) as a cluster with increased risk of dog ticks testing positive by PCR for the agent of tularemia. The cluster is approximately 290 meters in diameter and consists of 9 waypoints, fewer than 10% of the total waypoints. Although only 35% (2462 of 7035) of the total number of ticks was collected from this cluster, it yielded 64% (163 of 254) of the PCR-positive ticks. Dog ticks collected from this cluster were more likely to test positive for *F. tularensis* DNA than those collected in the rest of the field site (relative risk (RR) = 3.3, P = 0.001). We conclude that the intersection of the transects may represent a microfocus where the agent of tularemia stably perpetuates.

**Figure 4:**
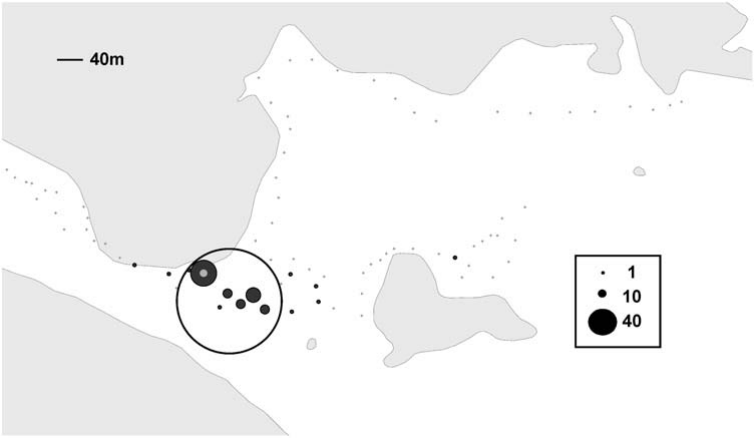
GIS mapping of the total number of positive ticks with uncommon VNTR haplotypes from each waypoint. The size of the dot at each waypoint is proportional to the number of ticks at each site. The location of the cluster identified by SaTScan is circled. Waypoint 70 is indicated by the gray dot.

**Figure 5:**
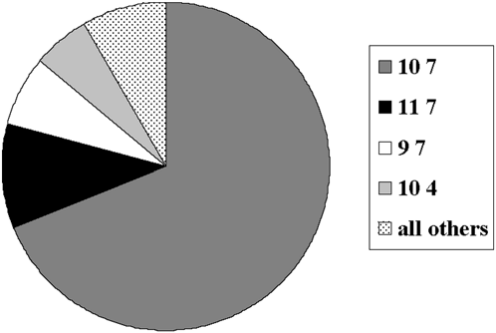
The distribution of the 13 identified VNTR haplotypes as a proportion of the total.

Over the 4 years of our study, we found 254 ticks that tested positive for *F. tularensis* DNA by PCR. VNTR analysis of the *F. tularensis* DNA from each of these demonstrated that ticks in our study site contained 13 unique haplotypes. The majority of positives (69%) comprised one haplotype, 10 7 (Figure 5). The remaining 31% (85 ticks) comprised 12 different haplotypes, which we designate “uncommon” haplotypes. The vast majority (84% of 85) of ticks with uncommon haplotypes derived from the cluster identified in the SaTScan analysis (Figure 4). Positive ticks collected in the cluster were 3.4 times (RR = 3.4, P<0.0001) more likely to have an uncommon haplotype than those collected elsewhere on the transect. Our identification of a microfocus by cluster analysis of prevalence is supported by the relative frequency of genetic variants in the same cluster.

Although SaTScan did not discriminate it as a separate cluster, there is one waypoint within the microfocus that appears to yield more infected ticks than other waypoints (denoted by a light gray dot on Figures 2–4) during the full 4 year study. This one waypoint, #70, not only yielded the most ticks for any single waypoint (10.5% of the total collected), but it also disproportionately provided more ticks testing positive for *F. tularensis* DNA (23.4%), than any other single waypoint. Table 2 compares the top 3 waypoints that were characterized by evidence of apparently intensive enzootic transmission. Waypoints 65 and 67 yielded rates of PCR-positive ticks proportional to the number of ticks collected at each site. Waypoint 70 yielded twice the expected number of PCR-positive ticks compared to the other two sites (RR = 2, P<0.05 for either comparison). Although the microfocus that we have identified is nearly 300 m in diameter, waypoint 70 appears to mark a discrete patch of ground within the microfocus that consistently generates infected ticks. We conclude that the perpetuation of the agent of tularemia may be extremely focal, even on the order of 7 meters or so in radius (the discrimination capacity of the GPS unit).

**Table 2:** Comparison of the three “hottest” waypoints.

Waypoint	% Total Ticks	% PCR-Positive	RR (p-Value)
70	10.5%	23.4%	-
65	7.8%	8.4%	2.08 (0.002)
67	4.2%	4.8%	2.00 (0.02)

The relative risk for the collection of PCR-positive ticks and collection of ticks harboring uncommon haplotypes was calculated for waypoint 70 vs. the other two waypoints.

## Discussion

The perpetuation of the agent of tularemia remains incompletely described, as is that of many other vector-borne infections. Type A tularemia (due to *F. tularensis tularensis*) has been argued to depend upon transmission cycles involving cottontail rabbits [11] and the ticks that feed upon them, and since its recognition as a zoonosis in the early 1900s, most human cases have been attributed to processing hunted or food production rabbits. Within the last 40 years, about half of the reported cases for which exposure data is known are due to tick exposure [12]. Although many different kinds of hematophagous arthropods are competent vectors in the laboratory, only ticks (*Dermacentor andersoni* and *D. variabilis*) and tabanid flies (*Chrysops* spp.) have been demonstrated to be important vectors to humans. Transovarial transmission appears to occur in ticks, although the literature varies with respect to this finding [13],[14]. Diverse animals have been reported to be infected [11] but other than rabbits, none have been definitively identified as a critical reservoir. Indeed, the role of animal reservoirs is arguable because a large proportion of species appear to die quickly from infection, suggesting transient intense epizootics followed by long periods of low enzootic activity as susceptible cohorts are renewed. It is likely that the perpetuation of *F. tularensis* in North American sites is due to a combination of horizontal (infection of various vertebrates, which in turn infect new ticks or flies) and vertical (infection of tick progeny transovarially) transmission [15]. Perpetuation occurs mainly in natural foci, where the pathobiocenosis is optimal. Epizootics occur due to unusual demographic events such as increased host density.

Although natural foci for tularemia have been reported, such foci comprise a vastly larger scale on the order of counties or the equivalent [10] and would be relatively useless for detailed studies of perpetuation of the agent. By means of a 4 year longitudinal study that mapped the location of dog ticks testing positive for *F. tularensis* DNA, we were able to identify a microfocus of transmission approximately 290 m in diameter or just less than 10% of our site. It is well established that ticks (even those that are not nest parasites) are not uniformly distributed across the landscape as a function of microhabitat requirements and host availability [16],[17]. Even within a site where microhabitat or hosts are homogenous, ticks infected by agents as diverse as rickettsiae, borreliae, or piroplasms occur in clusters [18]–[20]. Not surprisingly, we found that ticks containing *F. tularensis* DNA were clustered as well. What was surprising was that one cluster was so discrete and continuously demonstrable.

The use of VNTR analysis has greatly enhanced molecular epidemiology. Since the description of useful markers for *F. tularensis*, VNTR analysis has been used to demonstrate common exposures for cases of Type B tularemia [21],[22]. In addition, a fatal human tularemia case from the Martha's Vineyard outbreak yielded an isolate with a VNTR profile matching that which we reported from ticks near our study site, indicating a possible location of the exposure [22]. VNTR analysis is nicely complemented by GIS for detailed spatial analyses of transmission. (e.g., [23]). In our study, the complementary techniques have allowed us to definitively document a stable microfocus for *F. tularensis* transmission, an achievement that heretofore has been impossible. We found that ticks from the microfocus were more than 3 times more likely to contain *F. tularensis* characterized by uncommon VNTR haplotypes than those collected in the rest of our field site. The major method by which new VNTR haplotypes arise is by slip-strand mispairing of the tandem repeats [24]. The frequency with which this occurs depends on the number of repeats; in general the more repeats there are the more likely for mispairing to occur [25]. Of greater significance is that such mutations depend on the number of replication cycles the microbe undergoes, because each cycle provides an opportunity for mispairing to occur. Therefore, by mapping where ticks containing *F. tularensis* DNA with uncommon VNTR haplotypes are found, we were able to determine where increased replication ( = more intense transmission) is occurring. Alternatively, it is possible that the microfocus represents a more longstanding stable site of transmission. Although VNTR mutation rates have been established for certain bacteria such as *E. coli* [25] and *Yersinia pestis* [23], such a rate is speculative for *F. tularensis* except for the report that the 25 VNTR panel is invariant for 55 culture passages [21]. Thus, we cannot infer the age of our microfocus.

A panel of 25 VNTR markers can be used to characterize isolates of *F. tularensis* subspecies [26]. Although the use of all 25 markers might be more definitive with respect to describing the population structure of *F. tularensis*, we have found that the use of only two selected markers is sufficient for the objectives of our work. Indeed, we initially tested 6 of the most variable loci reported by Johansson et al. [26] on a subset of our samples and found that only Ft-M3 and Ft-M10 are easily amplifiable and variable for *F. tularensis* in our ticks. Most of the other markers did not vary for these samples and in fact one locus (Ft-M2) did not reproducibly amplify (unpublished data). In addition, because our DNA extracts derive from drops of tick hemolymph, we have a limited amount of template. The comprehensive 25 marker VNTR panel could only be used on the comparatively larger amount of DNA available from cultures or autopsy materials.

As with tick-borne encephalitis virus in *Ixodes ricinus* ticks, the agent of tularemia may persist in stable microfoci within permanent elementary foci [9],[27]; a small discrete area will yield infected ticks year after year. Unlike TBE virus, which might not perpetuate in the absence of ticks or animal hosts, it is possible that *F. tularensis* may persist as a fomite (nest materials, soil or water; even free-living protozoa; [28] given its alleged environmental stability [29]. Such fomites could theoretically lead to external contamination of ticks; however, because our assays focused on hemolymph, ticks that we considered to be positive by PCR represent those containing DNA as a result of biological transmission as opposed to those externally contaminated with bacteria. Tularemia microfoci may be unusually persistent given the possibility of environmental fomites, transovarial and transstadial transmission within ticks, as well as stable infection of diverse hematophagous arthropods, including fleas, mites, and lice [15].

The dog tick life cycle is well described [30]. Subadult ticks feed on small rodents such as voles and mice, although other animals such as rabbits, may sometimes be infested. Reproduction (feeding of adult female ticks) depends on medium sized mammals such as skunks, raccoons, foxes or coyotes. In many sites before the advent of acaricidal topical treatment, domestic dogs could serve as the main reproductive host in the absence of wild animals. Thus, for host-seeking adult ticks to contain *F. tularensis* DNA, infection must have been acquired either during subadult bloodmeals (transstadial transmission) or the bacteria passed by inheritance (transovarial transmission). A microfocus might be generated by chronic deposition of infected replete female ticks which would pass infection to their progeny (which would not disperse far on their own), but the finding of most of the uncommon bacterial haplotypes in the microfocus is difficult to reconcile with this hypothesis. Most ticks tend to contain a sole haplotype although a small proportion may contain two haplotypes (Goethert, unpublished).

We found ticks containing *F. tularensis* DNA everywhere in our field site but did not find evidence of any other microfocus. It may be that infected ticks are dispersed from the microfocus into the surrounding habitat by diverse animal hosts. Larvae from eclosed eggs cluster where the eggs had been deposited by an engorged female tick, and are dispersed by infesting a small mammal (rodent or insectivore) that forages through that cluster. In addition, adult ticks are dispersed from sites where host-seeking is intense by the widely ranging medium sized mammals that serve as their hosts. Skunks, a main reproductive host, may range more than a mile within a single night [31]. Accordingly, one scenario to partially explain the current Martha's Vineyard outbreak is that unusual amplification in microfoci, or dispersal of ticks from microfoci due to great densities of raccoons or skunks, seed new temporary foci and thereby expand the local distribution of infected ticks. These new temporary foci coalesce, promoting more homogenous risk. As susceptible animals die of tularemia and tick demes diminish in density in such temporary foci, risk becomes more and more heterogeneous in space as these foci disappear.

Identification of a microfocus will allow us to intensively analyze its nature and attempt to identify the components of the pathobiocenose. The microfocus consistently provided good numbers of host-seeking dog ticks; other parts of our transects contained large numbers of ticks at some point during the study, but unlike the microfocus, tick densities were transient. This observation suggests that dog ticks are not only good indicators with which to identify intense enzootic transmission but also that dog ticks are critical to perpetuating the agent in nature. Preliminary analysis of the microfocus, however, does not reveal a “smoking gun” burrow or evidence of intensive skunk or raccoon activity. Small mammals are no more likely to be trapped there nor is there gross evidence of intensive rodent activity. Indeed, there is nothing in the habitat structure of the microfocus that appears to be unusually conducive for transmission. It is likely that microhabitat-related factors (temperature, humidity, soil composition or chemistry, protozoal fauna, longstanding fomites) may serve as the basis for the microfocus.

## Materials and Methods

Tick collection: Host-seeking *Dermacentor variabilis* were collected by dragging monthly from April to August 2004–2007 in a privately owned, undeveloped area near Squibnocket on Martha's Vineyard, Massachusetts. This site comprises a morainal deposit with a scrubby open landscape. Impenetrable stands of scrub oak, bayberry, salt spray rose, poison ivy, blackberry, and high bush blueberry surround boggy, variably wet areas; the southern edge of the site is typical beach dune habitat with low-lying grasses and forbs. The property was used for sheep pasture in the 1800s and early 1900s but has otherwise not been developed. Pest infestations of dog ticks were well known from this site 80 years ago; it was included in a seminal tick control project using chalcid wasps undertaken by S. Burt Wolbach and Marshall Hertig of Harvard Medical School in the 1920s and 1930s [32]; (Telford, unpublished). The site contains a typical mammal fauna for coastal New England, including white footed mice, meadow voles, short tailed shrews, muskrats, cottontail rabbits, skunks and raccoons. The site is intensely infested by deer ticks (*Ixodes dammini* [ = *I. scapularis*]) which maintain the agents of Lyme disease, babesiosis, and human granulocytic ehrlichiosis.

A handheld GPS unit (Garmin Geko 201, WAAS enabled, with capacity to discriminate within 7 meters) was used to divide the field site into 85 waypoints along 3 transects (Figure 1). The total length of the 3 transects is roughly 3 km and they traversed the range of habitats within the site. Transects were drawn along existing roads and deer trails to minimize the impact on fragile habitat. Ticks were collected by flagging the vegetation along the transects and placing all ticks from a single waypoint together in one collection vial. A GPS location was thereby recorded for all the ticks collected from the field site.

PCR: PCR was used to test the ticks for evidence of *F. tularensis* infection as described previously [4]. Briefly, a drop of hemolymph was obtained from each of six ticks and placed in a tube with 50 ul of PBS. All ticks were then held in individual tubes labeled with the GPS site of their collection. The hemolymph pools were boiled for 15 minutes and used directly as template. A nested PCR directed against the fopA gene was used to screen for specific DNA as described previously. Ticks from pools testing positive were then reexamined individually. A second hemolymph test was performed if the tick was still alive, or if the tick was already dead, DNA extracts of whole tick homogenates were used (Qiagen DNeasy kit).

Variable number tandem repeat (VNTR) analysis was done using 2 loci as described previously [4]. Ft-M3 (previously called SSTR9) and Ft-M10 (previously called SSTR16) were amplified using a high fidelity Taq polymerase (Picomaxx, Stratagene). The forward primer of each was labeled with a fluorescent tag. Accurate sizing of the PCR amplicons was obtained by running them with the ABI500 (Applied Biosystems) markers on a capillary sequencer using Genemapper software (University of Maine Sequencing Facility, Orono, ME). The measured peaks evident from the amplicons were then analyzed using STRand (Veterinary Genetics Lab, University of California) or Peak Scanner Software v1.0 (Applied Biosystems). Haplotype designations are simply the number of repeating units for Ft-M3 followed by Ft-M10.

Prevalence and binomial confidence intervals were calculated using the statistical calculator at http://statpages.org/confint.html. Odds ratios, relative risks and Chi-squared tests of significance were obtained using the calculator at http://statpages.org/ctab2x2.html.

GIS analysis: The transect waypoints were mapped using using ArcMap software (ESRI ArcMap v9.1) and overlayed onto a Massachusetts islands watershed datalayer (MASSGIS http://www.mass.gov/mgis/laylist.htm). We used SaTScan 7.0 [33] for cluster analysis of our data. This program is freeware designed specifically for the spatial or space-time analysis of disease clusters and has been used in many similar studies [34]–[36]. SaTScan uses a circular window that moves through space to identify clusters. The window varies in size up to 50% of the population tested allowing for the identification of small and large clusters. A likelihood ratio test is done to determine whether an area has an elevated rate of “cases” compared to its surroundings. Significance (set a priori at P<0.05) is then calculated using Monte Carlo replicates.
